# Policy responses to viral hepatitis B and C among people who inject drugs in Member States of the WHO European region: a sub-analysis of the WHO 2013 global hepatitis policy survey

**DOI:** 10.1186/1471-2334-14-S6-S15

**Published:** 2014-09-19

**Authors:** Alexander Spina, Irina Eramova, Jeffrey V Lazarus

**Affiliations:** 1 Centre for Infectious Disease Epidemiology, Austrian Agency for Health and Food Safety (AGES), Vienna, Austria; 2 World Health Organization Regional Office for Europe, Copenhagen, Denmark; 3 CHIP, Centre for Health and Infectious Disease Research and WHO Collaborating Centre on HIV and Viral Hepatitis, Rigshospitalet, University of Copenhagen, Copenhagen, Denmark

## Abstract

**Background:**

Unsafe injections, through infectious bodily fluids, are a major route of transmission for hepatitis B and C. Viral hepatitis burden among people who inject drugs is particularly high in many Member States of central and Eastern Europe while national capacity and willingness to address it varies greatly.

In 2013, the World Health Organization conducted a survey assessing national viral hepatitis efforts of 194 national governments. Here, we present a sub-analysis of this global survey focusing on questions relating to people who inject drugs in the WHO European Region.

**Methods:**

The initial survey included 43 questions covering awareness, data, prevention, and screening and treatment. It was sent in five languages to identified national focal points. This sub-analysis included 11 questions and 53 Member States in the WHO European Region. Descriptive analyses of national activities are presented. As a secondary outcome bivariate analyses of differences between Member States of the European Union (EU) and European Free Trade Association (EFTA) compared to those not in said grouping are presented.

**Results:**

Forty-four of the 53 Member States responded to the survey (response rate of 83%). More than three-quarters reported offering publicly-funded treatment for HBV or HCV (82% and 80%, respectively), with a significantly higher proportion of EU/EFTA Member States (P=0.004 and P=0.010, respectively). Half of Member States (53%) reported the existence of a national policy for hepatitis prevention and control; however less than one-third (27%) reported having written national strategies. Under half of the responding Member States reported holding events for World Hepatitis Day 2012. One-fifth reported offering hepatitis B and C testing free of charge, with less than one-third reportedly conducting regular serosurveys among people who inject drugs.

**Conclusions:**

Findings highlight key gaps requiring attention in order to improve national policies and programmes in the region and ensure an adequate response to injection drug use-associated viral hepatitis. Further studies are required to assess quality and impact of national policies and services.

## Introduction

Hepatitis B and C (HBV and HCV) are transmitted through exposure to infectious bodily fluids. Transmission may be through infected blood products or transfusions as well as contaminated injections in medical settings and among people who inject drugs (PWID).

Hepatitis B and C can produce acute illness ranging from asymptomatic to fatal. Approximately 90% of HBV infections are transient and patients recover fully [1]. In persistent cases chronic infections occur, with patients remaining infectious and developing liver cirrhosis, hepatocellular carcinoma or both over a period of several decades [2,3]. Modelling suggests that there were 240 million HBV surface antigen (HBsAg) positive persons in 2005 [4] and more than 185 million people were HCV antibody-positive (anti-HCV) in 2010 [5]. Positive HBsAg corresponds to current infection, with antibodies to the HBV core antigen (anti-HBc) and anti-HCV corresponding to current or past infection for HBV and HCV, respectively [2,3]. According to Global Burden of Disease estimates, HBV and HCV together caused 1.3 million deaths in 2010 [6];on par with other major infectious diseases including malaria (causing 660,000 deaths in 2010) [7], tuberculosis and HIV (causing 1.4 and 1.7 million deaths in 2011, respectively) [8,9].

A safe and effective vaccine is available for hepatitis B but not for hepatitis C. Both can be treated with antiviral agents. Treatment for hepatitis B infection has been shown to reduce the risk of hepatocellular carcinoma and thereby mortality rates[10]. Hepatitis C is widely regarded as curable, but treatment access barriers prevent many people from benefiting from the available treatments [11,12].

Unsafe injections, especially in the context of non-medical injection drug use, contribute disproportionately to the transmission of HBV and HCV [13]. This is particularly the case in Eastern Europe and Central Asia [14,15].

A 2011 study reported approximately ten million PWID were anti-HCV positive and 6.4 million were positive for anti-HBc with 1.2 million being HBsAg-positive globally. The largest populations of HBsAg and HCV-positive PWID were found to live in eastern Europe: 0.3 and 2.3 million, respectively [16].

A recent study reported PWID had the highest prevalence of HBC and HCV infections in countries of the WHO European region outside of the European Union/European Free Trade Association (EU/EFTA) [17]. Outside of the EU/EFTA, 21% of PWID were estimated to be infected with HBV, while HBV prevalence in the EU/EFTA was 3.7%. HCV prevalence in the EU/EFTA and non-EU/EFTA regions were 47% and 43% respectively, indicating that even in higher income European countries, injection drug use leads to high prevalence rates of HCV.

Another study estimated that injection drug use as a risk factor for hepatitis C accounted for 502,000 disability-adjusted life years [18]. The 2013 European Drug Report suggests the problem may be increasing in lower-income states of the EU/EFTA [19]. Countries in eastern Europe and central Asia have seen an increase in the population of PWID as well as an accelerating HIV epidemic [20]. Increases in HIV co-infection with HBV and HCV among PWID in these countries have led to viral hepatitis becoming a major cause of death among HIV-positive people [21]. Co-infection of HCV with tuberculosis is also common among PWID, with two thirds who develop tuberculosis also being anti-HCV positive [12]. In response, the European Union adopted a Written Declaration on hepatitis B and C on 18 November 2013 calling for the recognition of these diseases as an urgent public health issue requiring priority actions [22].

In order to take stock of national policy responses to viral hepatitis worldwide, the World Health Organization (WHO) in collaboration with the World Hepatitis Alliance and the University of Copenhagen conducted a survey of 194 WHO Member States in 2012–2013. This study presents a sub-analysis of survey data from the Member States of the WHO European Region, focusing particularly on policy issues and activities that are relevant to the prevention and control of viral hepatitis among people who inject drugs.

## Methods

The WHO hepatitis policy survey contains 43 questions, most of which request information relating to the four strategic axes outlined in the WHO Global Hepatitis Programme Framework for Global Action (Table 1) [23]. The survey was written in English and piloted in 13 WHO Member States in 2012. Following the incorporation of feedback, the survey was translated into French, Portuguese, Russian and Spanish. The final version of the survey was distributed to identified focal points for viral hepatitis in national ministries, departments of health or related entities in 194 Member States. Selection of these focal points was guided by direct communication with government agencies as well as by input from WHO regional and country offices.

**Table 1:** Strategic axes outlined in the Global Hepatitis Programme framework

Axis	Objective
Axis 1	Raising Awareness, Promoting Partnerships and Mobilizing Resources

Axis 2	Evidence-Based Policy and Data for Action

Axis 3	Prevention of Transmission

Axis 4	Screening, Care and Treatment

Data collection took place between July 2012 and April 2013. Survey responses were e-mailed to study coordinators at the University of Copenhagen, who compiled a dataset of responses. Findings were reported in the *Global policy report on the prevention and control of viral hepatitis* in 2013 [24].

For the purpose of this sub-study, eleven survey questions relevant to PWID were selected (Additional file 1). The analysis utilised survey responses of 53 Member States comprising the WHO European Region. These Member States were categorised as EU/EFTA and non-EU/EFTA states for comparison. Thirty-one Member States fell within the EU/EFTA area.^1^ Two additional Member States under monetary agreements with the EU, Andorra and San Marino, were included in the subgroup given their geographic and socioeconomic proximity. Twenty Member States were non-EU/EFTA states.^2^

Descriptive statistics were created for the activities that Member States reported in the survey. A secondary analysis compared the aforementioned subgroupings. As a basic indication of differences between the EU/EFTA and non-EU/EFTA regions, bivariate analysis using χ^2^ testing was performed. Bivariate analyses were two-tailed with a significance level of 0.05. All subgroup findings are reported, and subgroup differences that achieved statistical significance are reported with the p-value. An additional bivariate analysis compared the World Bank income classifications of countries in the EU/EFTA and non-EU/EFTA regions, using July 2012 definitions of high-income, upper-middle-income, lower-middle-income and low-income countries [25]. All analyses were carried out using IBM SPSS version 20.0 software (Armonk, NY, USA).

## Results

### Respondents

Forty-four of the 53 Member States in the WHO European region replied to the survey and were included in the analysis, a response rate of 83%. Twenty-eight of the 33 EU/EFTA Member States (85%) responded in comparison to 16 of 20 (80%) non-EU/EFTA Member States. The majority (89%) of responding EU/EFTA Member States were high-income countries, while only 6% of non-EU/EFTA respondents were high-income countries (p<0.001) (Table 2).

**Table 2:** Distribution of national income by EU/EFTA membership

	Non EU/EFTA n=16 (%)	EU/EFTA n=28 (%)	P-value
**High income**	1 (6)	25 (89)	<0.001*
	
**Upper middle-income**	7 (44)	3 (11)	
	
**Lower middle-income**	5 (31)	0 (0)	
	
**Lower income**	3 (19)	0 (0)	

Table 3 reports the number and percentage of Member States responding affirmatively to each survey question included in this analysis.

**Table 3:** Governments in the WHO European Region reporting activities addressing viral hepatitis by WHO Global Hepatitis Programme strategic axis (N=44)

		**N=44** n (%)	Member States Reporting Activities
**National Coordination**	Have viral hepatitis programme activities targeting PWID as a specific population	32 (73)	Armenia, Austria, Belarus, Belgium, Bulgaria, Croatia, Czech Republic, Denmark, Finland, France, Ireland, Israel, Italy, Kyrgyzstan, Latvia, Luxembourg, Malta, Montenegro, Netherlands, Poland, Republic of Moldova, Russian Federation, San Marino, Serbia, Slovakia, Slovenia, Spain, Sweden, Switzerland, The Former Yugoslav Republic of Macedonia, Turkey, United Kingdom
	
	Have a written national viral hepatitis prevention and control strategy or plan which includes a component relating to injection drug use	12 (27)	Armenia, Austria, Czech Republic, Denmark, France, Israel, Kyrgyzstan, Republic of Moldova, Russian Federation, Slovenia, Turkey, United Kingdom

**Axis 1**	Held events for World Hepatitis Day 2012	17 (39)	Albania, Armenia, Azerbaijan, Belarus, Bulgaria, Croatia, France, Georgia, Italy, Latvia, Lithuania, Malta, Netherlands, Republic of Moldova, Russian Federation , Slovenia, Sweden
	
	Funded viral hepatitis awareness campaigns focusing on harm reduction for PWID (other than World Hepatitis Day)	10 (23)	Armenia, Belarus, Croatia, Netherlands, Republic of Moldova, Russian Federation, Slovenia, Sweden, Turkey, United Kingdom

**Axis 2**	Conduct regular serosurveys among PWID	12 (27)	Belarus, Croatia, Denmark, Finland, France, Montenegro, Moldova, Russian Federation, Slovenia, Tajikistan, Ukraine, United Kingdom

**Axis 3**	Have a national policy for prevention of viral hepatitis among PWID	25 (53)	Armenia, Austria, Belarus, Belgium, Bulgaria, Croatia, Czech Republic, Denmark, France, Israel, Italy, Kyrgyzstan, Luxembourg, Netherlands, Poland, Republic of Moldova, Russian Federation, Slovenia, Spain, Sweden, Switzerland, The Former Yugoslav Republic of Macedonia, Turkey, United Kingdom, Uzbekistan

**Axis 4**	Have HBV and HCV testing which is:		
	• Free of charge for all individuals	20 (45)	• Albania, Austria, Azerbaijan, Belarus, Belgium, Croatia, Denmark, Finland, France, Germany, Israel, Italy, Montenegro, Republic of Moldova, Malta, San Marino, Slovakia, Spain, Sweden, United Kingdom
	• Free of charge for PWID	6 (14)	• Bulgaria, Czech Republic, Latvia, Slovenia Turkey, The Former Yugoslav Republic of Macedonia
	
	Have publicly funded treatment for HBV	36 (82)	Andorra, Armenia, Austria, Belarus, Belgium, Bulgaria, Croatia, Cyprus, Czech Republic, Denmark, Estonia, Finland, France, Germany, Hungary, Ireland, Italy, Latvia, Lithuania, Luxembourg, Malta, Montenegro, Netherlands, Poland, Republic of Moldova, Russian Federation, San Marino, Serbia, Slovakia, Slovenia, Spain, Sweden, The Former Yugoslav Republic of Macedonia, Turkey, United Kingdom
	
	Have publicly funded treatment for HCV	35 (80)	Andorra, Armenia, Austria, Belarus, Belgium, Bulgaria, Croatia, Cyprus, Czech Republic, Denmark, Estonia, Finland, France, Germany, Hungary, Ireland, Israel, Italy, Latvia, Lithuania, Luxembourg, Malta, Montenegro, Netherlands, Poland, Republic of Moldova, Russian Federation, San Marino, Serbia, Slovenia, Spain, Sweden, The Former Yugoslav Republic of Macedonia, Turkey, United Kingdom

### National coordination

Twelve responding Member States (27%) reported having a written national prevention and control strategy which addressed injecting drug use. Six each of non-EU/EFTA and EU/EFTA Member States (38% and 21%, respectively) reported a written national strategy for injection drug use (Table 4).

**Table 4:** Governments in the WHO European region reporting activities addressing viral hepatitis by WHO Global Hepatitis Programme strategic axis and EU/EFTA membership (N=44)

		**Non EU/EFTA n=16** (%)	EU/EFTA n=28 (%)	P-value
**National coordination**	Have viral hepatitis programme activities address PWID as a specific population	10 (63)	22 (79)	0.512
	
	Have a written national viral hepatitis prevention & control strategy which also targets ID use	6 (38)	6 (21)	0.335

**Axis 1**	Held events for World Hepatitis Day 2012	7 (44)	10 (36)	0.765
	
	Funded viral hepatitis awareness campaigns focusing on harm reduction for PWID (other than World Hepatitis Day)	5 (31)	5 (18)	0.404

**Axis 2**	Conduct regular serosurveys among PWID	6 (38)	6 (21)	0.639

**Axis 3**	Have a national policy for prevention & control of viral hepatitis among PWID	9 (56)	16 (57)	0.954

**Axis 4**	Have HBV & HCV testing which is:			
	• Free of charge for all	6 (38)	14 (50)	0.569
	• Free of charge for PWID	2 (13)	4 (14)	0.423
	
	Have publicly funded treatment for HBV	9 (56)	27 (96)	0.004*
	
	HBV Drugs on national essential medicines list or subsidized by government:			
	• Interferon alpha	12 (75)	22 (79)	0.786
	• Pegylated interferon	11 (69)	22 (79)	0.469
	• Lamivudine	13 (81)	24 (86)	0.697
	• Adefovir dipivoxil	4 (25)	20 (71)	0.003*
	• Entecavir	3 (20)	19 (68)	0.002*
	• Telbivudine	4 (25)	13 (46)	0.160
	• Tenofovir	8 (50)	19 (68)	0.242
	
	Have publicly funded treatment for HCV	9 (56)	26 (93)	0.010*
	
	HCV Drugs on national essential medicines list or subsidized by government:			
	• Interferon alpha	8 (50)	22 (79)	0.050*
	• Pegylated interferon	12 (75)	23 (82)	0.572
	• Ribavirin	14 (88)	24 (86)	0.868
	• Boceprevir	2 (13)	15 (54)	0.007*
	• Telaprevir	3 (19)	14 (50)	0.041*

Thirty-two responding Member States (73%) reported having some form of national prevention and control programme which included activities addressing PWID as a specific population. Ten non-EU/EFTA Member States (69%) and 22 EU/EFTA Member States (79%) reported programme activities addressing PWID.

### Awareness-raising and evidence to guide policy

In the WHO European Region 17 Member States of the 44 (39%) who responded to the survey reported holding events for World Hepatitis Day (WHD); of these, 7 Member States and an additional 3 Member States, that did not hold events for WHD, reported organizing viral hepatitis awareness campaigns (28%), other than WHD, with messages on harm reduction. The number of responding Member States reporting activities for WHD was higher, 10 (36%), in the EU/EFTA subgrouping, however a higher proportion of non EU/EFTA Member States reported activities, 7 (44%). Similarly the proportion of non EU/EFTA Member States reporting awareness activities other than WHD was higher than EU/EFTA, with 8% compared to 31%.

In the WHO European region, 12 responding Member States (27%) reported conducting regular sero-surveys among PWID; six each from EU/EFTA (21%) and non-EU/EFTA (38%) subgroupings.

### Prevention, testing and treatment

Twenty-five responding Member States (53%) reported having a national policy for prevention of viral hepatitis among PWID; nine (56%) of which were non-EU/EFTA and 16 (57%) EU/EFTA. With regards to testing for HBV and HCV, 20 (45%) Member States reported having universal testing free of charge, while 6 (14%) other Member States reported having testing free of charge for PWID. Six (38%) non-EU/EFTA and 14 (50%) EU/EFTA Member States reported universal testing free of charge, with 2 (13%) and 4 (14%) reporting testing free of charge for PWID.

Thirty-six (82%) responding Member States reported having publicly funded HBV treatment. A significantly higher proportion (p=0.004) of EU/EFTA Member States compared to non-EU/EFTA Member States reported having publicly funded treatment for HBV with 27 (96%) compared to 9 (56%), respectively. Proportions of Member States in the two subgroupings reporting drugs subsidised or on the national essential-medicines list were similar for several drugs; however Adefovir-dipivoxil and Entecavir were reported more often (p=0.002) by EU/EFTA Member States (20 (71%) and 19 (68%), respectively) compared to non-EU/EFTA Member States (4(25%) and 3(20%), respectively).

Thirty-five (80%) responding Member States reported having publicly funded treatment for HCV. A significantly higher proportion (p=0.010) of EU/EFTA Member States reported having publicly funded treatment for HCV, 26 (93%), compared to non-EU/EFTA Member States, 9 (56%). Significantly more EU/EFTA Member States than non-EU/EFTA Member States reported having interpheron alpha (p=0.050), Boceprevir (p=0.007) and Telaprevir (p=0.041); with 22 (79%) compared to 8 (50%), 15 (54%) compared to 2 (13%) and 14 (50%) compared to 3 (19%), respectively. Two Member States reported only funding treatment for acute infections with two further Member States only partially subsidising chronic treatment [data not shown] for both HCV and HBV.

## Discussion

This study is a sub-analysis of the WHO Global Hepatitis Policy Survey, focusing on the countries of the WHO European Region. It provides crucial insight into a central public health problem in the region: very high prevalence of viral hepatitis among PWID. Given the extent of the problem, and a previous reticence to address it, it was encouraging to note that Member States in the region are willing to engage with WHO in order to address viral hepatitis.

Various means of addressing viral hepatitis among PWID at national and global levels exist. A WHO guidance document presented five recommendations on the prevention of viral hepatitis B and C among PWID in 2012 [26]. These included offering a rapid HBV vaccination regimen, offering incentives to increase uptake and completion of the HBV vaccination schedule, suggestions that needle and syringe programmes provide low dead-space syringes for distribution to PWID, not using psychosocial interventions for PWID and instead to offer peer interventions for reduction of viral hepatitis incidence. These recommendations can be categorised into the four strategic axes of the WHO Global Hepatitis Programme (Table 1) outlined in the Framework for Global Action [23]. It is essential that policy responses encompass aspects of these four strategic areas.

A notably positive finding is that over half of responding Member States reported having a national policy for the prevention of viral hepatitis among PWID, as compared to 37% globally [24]. A higher proportion of Member States reported having programme activities addressing viral hepatitis among PWID; seven Member States reported activities even in the absence of national policies. Less promising is the finding that less than one-third of Member States reported having written national viral hepatitis prevention and control strategies that include a component addressing PWID. Such documents are the foundation for strong, evidence-based, national response to the complex array of factors contributing to the hepatitis epidemic among PWID. Further investigation is required to assess the structure and content of national programme activities and other efforts, including the work of nongovernmental organisations.

In light of new drug therapies, it is particularly promising that more than 80% of Member States offer publicly funded treatment for HBV and HCV. Experts predict that in the next 2-5 years, 90% of HCV infections will be curable with an all-oral, once-daily, 12-week regimen of safe drugs as compared with the current regimen requiring 24 to 48 weeks of weekly injections and results in cure-rates of 45-80% [27]. Indeed, oral only treatment attained a sustained viral response in 8 weeks in a trial of high-risk HCV-patients [28], promising results for addressing adherence and treatment-cost. Recent WHO guidelines indicate treatment of HCV among PWID is efficacious, cost-effective and may be effective as prevention due to the reduction in transmission [12]. Chronic HBV therapy is also improving with drug regimens becoming more potent and easier to administer [29]. However, the persistence of treatment access barriers means that treatment uptake and adherence among PWID remains low [13,14,23]. Major barriers to treatment include stigmatisation and systematic exclusion of former or current PWID in some nations, along with criminalisation [13,14,30,31]. Indeed, one Member State reported that injection drug use is a contraindication for beginning treatment, with several Member States only funding acute treatment. Results should be interpreted with caution where governments fund non-specific treatment, which means treating symptoms rather than the disease. In situations where specific treatment is funded, further investigation is warranted to clarify whether treatment funding policies are comparable across the region. Gaps in funding specific treatment is exemplified by results from non-EU/EFTA Member States, where a large proportion report funding Interferons but not full treatment regimens for HCV; however differences in public funding may be attributable to national income distribution differences between EU/EFTA and not.

Given the low socio-economic status of many PWID, beyond funding treatment, government policies which support and encourage cost-effective prevention programmes, such as vaccination and needle exchange programmes, are essential [13,14,32]. These need to be supplemented by alternative intervention methods which have been shown to be effective. In example removing users from the PWID population [32] or transitioning to non-injecting drug use [33]. Coupling these with initiatives to enable access to affordable, quality screening and treatment is key to curtailing the epidemic among PWID. Prevention and access campaigns are increasingly important to control unsafe injection-associated infections, as the illicit nature and associated marginalisation of PWID populations make them hard to reach for conventional healthcare providers [14,34]. A recent review outlines the need for social interventions relating to housing, stigma reduction and systemic changes in policy and health care delivery in order to improve access and uptake of hepatitis C treatment among PWID [35]. Social change can be initiated through policy and legislation. Reports suggest that activities to address unsafe injection-associated infectious disease among PWID have been implemented to varying degrees by Member States in the region [9,14,24].

Epidemiological data detailing prevalence by population-groups is vital for both policy-makers and healthcare planners to assess intervention costs and ensure evidence-based planning of responses. Such data can be gathered, among other methods, from serosurveys as well as centralised reporting of screening results. Screening is also essential for identifying those who would benefit from treatment and thereby reduce the personally and monetarily, costly sequelae of infection [36,37]. Thus it is in the interest of policy makers to support this evidence base. High-quality data from hepatitis prevalence studies are sparse in Eastern Europe and Central Asia [38].

Findings regarding national serosurveys are worrying, with under one-third of Member States conducting them regularly. Similar to the ~40% that reported free testing, globally [24]. Surprisingly, a higher proportion, though not statistically significant, of non-EU/EFTA Member States reported conducting regular serosurveys; it would be expected that EU/EFTA members would have such surveillance methods in place, due to support by the European Centre for Disease Prevention and Control. It is important to present such data to policy-makers in order to ensure that decisions are taken based on representative data. Though not definitive, this lack of data may explain our finding of Member States reporting programme activities not being based on written strategies.

Less than half offer HBV and HCV testing free of charge; with a non-significantly higher proportion of EU/EFTA Member States reporting publicly funded testing. Funding differences may be attributable to the income distribution differences between EU/EFTA and non-EU/EFTA. Details of which screening tests were funded could not be gleaned from the survey and future studies may benefit from investigating this detail. Facilitating access to screening services is particularly pertinent when dealing with marginalised populations such as PWID.

Under two fifths of responding Member States reported holding events for World Hepatitis Day 2012 (28 July). This is troubling, as awareness-raising is such a vital first-step in controlling these diseases, particularly among PWID, where educational messages on harm reduction have been shown to be effective[19].

An argument could be made for policies to be focused on any of the four areas. However, as emphasis on one area may impact the remaining three, it is important that efforts in all four areas are scaled up in unison to address viral hepatitis effectively. A recent review details the need for a combination of preventative efforts to address worldwide burden [30].

Our study provides valuable information to policy-makers and health analysts in the WHO European region on the extent to which viral hepatitis is being addressed among PWID. It is anticipated that policy makers will utilise this information in order to compare progress of Member States in the region with the intent of identifying areas which could be improved and inform further investigation in to national programmes in their respective countries.

Several limitations may have influenced study findings. Survey questions sought to document the existence of national policies, strategies and programmes without seeking to assess the extent to which they are being implemented. The survey also did not yield information on quality or impact of programmes. To this end, further investigations need to be undertaken to verify Member States reports and to assess how policies, strategies and programmes are contributing to beneficial outcomes. Patient groups and other nongovernmental organisations have an important role to play in these investigations; this study could have benefited from input by said parties as well as civil society groups.

Linguistic and semantic considerations should also be highlighted. As the survey was limited to the aforementioned five languages, response rates and a thorough, clear understanding of survey questions may have been affected. Ambiguity as well as variations in the interpretation of questions by region or culture may have also led to differential understanding of, and responses to, survey questions.

In addition, data included here are those reported by identified focal-points from each Member State. It was not possible to verify the data submitted prior to writing this manuscript. Further studies are currently being undertaken by the WHO global hepatitis programme to assess the quality and content of national programmes. Bivariate analyses conducted are basic indications with many potential confounders and do not describe definitive or causal associations, particularly due to low sample-size. Further studies would benefit from investigating reasons behind differences in national policies and activities.

In summary, this sub-analysis highlights gaps which require attention, particularly with regards to the evidence base and awareness, in order to improve policies and programme activities addressing viral hepatitis among PWID in the European region. Further studies are urgently needed to detail areas requiring policy attention and to ensure an effective response to this global health problem.

## Competing interests

None to declare.

## Authors’ contributions

JVL led the initial data acquisition. AS and JVL developed the conception and design of the current study. AS carried out analysis and interpretation of data as well as drafted the manuscript. JVL and IE revised the manuscript critically to improve content. All authors read and approved the final manuscript.

## Notes

^1 ^**EU/EFTA Member States as of December 2013:** Austria, Belgium, Bulgaria, Croatia, Cyprus, Czech Republic, Denmark, Estonia, Finland, France, Germany, Greece, Hungary, Iceland, Ireland, Italy, Latvia, Lithuania, Luxembourg, Malta, Netherlands, Norway, Poland, Portugal, Romania, Slovakia, Slovenia, Spain, Sweden, Switzerland, United Kingdom of Great Britain and Northern Ireland.

^2 ^**Non-EU/EFTA:** Albania, Armenia, Azerbaijan, Belarus, Bosnia and Herzegovina, Georgia, Israel, Kazakhstan, Kyrgyzstan, Monaco, Montenegro, Republic of Moldova, Russian Federation, Serbia, Tajikistan, The Former Yugoslav Republic of Macedonia, Turkey, Turkmenistan, Ukraine, Uzbekistan

## Supplementary Material

Additional file 1 Survey instructions and questions selected for this sub-analysis presented with initial survey numbering Click here for file

## Declarations

The survey was managed at the University of Copenhagen with funding from the World Hepatitis Alliance. This article has been published as part of *BMC Infectious Diseases* Volume 14 Supplement 6, 2014: Viral Hepatitis in Europe. The full contents of the supplement are available online at http://www.biomedcentral.com/bmcinfectdis/supplements/14/S6. The publication charges for this supplement were funded by AbbVie as an unrestricted grant to Rigshospitalet, the University of Copenhagen. AbbVie further funded the printing of the supplement with additional financial support from the Drug Prevention and Information Programme (DPIP) of the European Union.

## References

[B1] Mahoney F Kane M Plotkin SA and Orenstein WA Hepatitis B Vaccine Vaccines 1999 3 Philadelphia: Saunders Company

[B2] Lee WM Hepatitis B Virus Infection N Engl J Med 1997 337 1733 1745 9392700 10.1056/NEJM199712113372406

[B3] Lauer GM Walker BD Hepatitis C virus infection N Engl J Med 2001 345 41 52 11439948 10.1056/NEJM200107053450107

[B4] Ott JJ Stevens GA Groeger J Wiersma ST Global epidemiology of hepatitis B virus infection: new estimates of age-specific HBsAg seroprevalence and endemicity Vaccine 2012 30 2212 2219 22273662 10.1016/j.vaccine.2011.12.116

[B5] Mohd Hanafiah K Groeger J Flaxman AD Wiersma ST Global epidemiology of hepatitis C virus infection: new estimates of age-specific antibody to HCV seroprevalence Hepatol Baltim Md 2013 57 1333 1342 10.1002/hep.26141 23172780

[B6] Lozano R Naghavi M Foreman K Lim S Shibuya K Aboyans V Abraham J Adair T Aggarwal R Ahn SY Alvarado M Anderson HR Anderson LM Andrews KG Atkinson C Baddour LM Barker-Collo S Bartels DH Bell ML Benjamin EJ Bennett D Bhalla K Bikbov B Bin Abdulhak A Birbeck G Blyth F Bolliger I Boufous S Bucello C Burch M Global and regional mortality from 235 causes of death for 20 age groups in 1990 and 2010: a systematic analysis for the Global Burden of Disease Study 2010 Lancet 2012 380 2095 2128 23245604 10.1016/S0140-6736(12)61728-0 PMC10790329

[B7] World Health Organization | World Malaria Report 2012 Geneva, Switzerland http://www.who.int/malaria/publications/world_malaria_report_2012/en/

[B8] World Health Organization | Global Tuberculosis Report 2012 Geneva, Switzerland http://www.who.int/tb/publications/global_report/en/

[B9] UNAIDS | 2012 UNAIDS Report on the Global AIDS Epidemic Geneva http://www.unaids.org/en/resources/publications/2012/name,76121,en.asp

[B10] Dienstag JL Hepatitis B virus infection N Engl J Med 2008 359 1486 1500 18832247 10.1056/NEJMra0801644

[B11] Poynard T Yuen M-F Ratziu V Lai CL Viral hepatitis C Lancet 2003 362 2095 2100 14697814 10.1016/s0140-6736(03)15109-4

[B12] WHO | Guidelines for the screening, care and treatment of persons with hepatitis C infection http://www.who.int/hiv/pub/hepatitis/hepatitis-c-guidelines/en/25535634

[B13] Robaeys G Grebely J Mauss S Bruggmann P Moussalli J Gottardi AD Swan T Arain A Kautz A Stöver H Wedemeyer H Schaefer M Taylor L Backmund M Dalgard O Prins M Dore GJ Recommendations for the Management of Hepatitis C Virus Infection Among People Who Inject Drugs Clin Infect Dis 2013 57 suppl 2 S129 S137 23884061 10.1093/cid/cit302

[B14] Global Commission on Drug Policy | The Negative Impact of The War On Drugs On Public Health: The Hidden Hepatitis C Epidemic Rio de Janeiro, Brazil http://www.globalcommissionondrugs.org/hepatitis/

[B15] European Centre for Disease Prevention and Control | Hepatitis B and C in the EU neighbourhood: prevalence, burden of disease and screening policies Stockholm, Sweden http://ecdc.europa.eu/en/publications/Publications/Forms/ECDC_DispForm.aspx?ID=560

[B16] Nelson PK Mathers BM Cowie B Hagan H Des Jarlais D Horyniak D Degenhardt L Global epidemiology of hepatitis B and hepatitis C in people who inject drugs: results of systematic reviews Lancet 2011 378 571 583 21802134 10.1016/S0140-6736(11)61097-0 PMC3285467

[B17] Hope VD Eramova I Capurro D Donoghoe MC Prevalence and estimation of hepatitis B and C infections in the WHO European Region: a review of data focusing on the countries outside the European Union and the European Free Trade Association Epidemiol Infect 2013 1 17 10.1017/S0950268813000940 PMC3891474 23714072

[B18] Degenhardt L Whiteford HA Ferrari AJ Baxter AJ Charlson FJ Hall WD Freedman G Burstein R Johns N Engell RE Flaxman A Murray CJL Vos T Global burden of disease attributable to illicit drug use and dependence: findings from the Global Burden of Disease Study 2010 Lancet 2013 382 1564 1574 23993281 10.1016/S0140-6736(13)61530-5

[B19] European Monitoring Centre for Drugs and Drug Addiction | European Drug Report 2013: Trends and developments Lisbon, Portugal http://www.emcdda.europa.eu/publications/edr/trends-developments/2013 23787082

[B20] Hickson F Latypov A Reynolds L Platt L Hope V Jolley E Rhodes T HIV in the European Region: Using Evidence to Strengthen Policy and Programs 2013 The World Bank 1 20

[B21] World Health Organization Executive Board| Viral hepatitis report by the Secretariat. EB126/15 Geneva, Switzerland http://apps.who.int/gb/ebwha/pdf_files/EB126/B126_15-en.pdf

[B22] Ticau S-A Octavia-Sarbu D Sorin-Ivan C Tanasecu CC Cretu C Tarabella M Gierek A Rosbach A Carvalho MDG Grossetete francoise Paravanova A Written Declaration submitted under Rule 123 of the Rules procedure on hepatitis B and C 2013

[B23] World Health Organization| Prevention and Control of Viral Hepatitis Infection: Framework for Global Action Geneva, Switzerland http://www.who.int/csr/disease/hepatitis/Framework/en/

[B24] World Health Organization | Global policy report on the prevention and control of viral hepatitis Geneva, Switzerland http://www.who.int/csr/disease/hepatitis/global_report/en/

[B25] Country and Lending Groups | Data http://data.worldbank.org/about/country-classifications/country-and-lending-groups

[B26] World Health Organization | Guidance on prevention of viral hepatitis B and C among people who inject drugs Geneva, Switzerland http://www.who.int/hiv/pub/guidelines/hepatitis/en/23805439

[B27] Liang TJ Ghany MG Current and Future Therapies for Hepatitis C Virus Infection N Engl J Med 2013 368 1907 1917 23675659 10.1056/NEJMra1213651 PMC3893124

[B28] Kohli A Sims Z Nelson A Combination oral hepatitis C antiviral therapy for 6 or 12 weeks: final results of the SYNERGY trial 2014 Boston

[B29] Lai C-L Yuen M-F Chronic Hepatitis B — New Goals, New Treatment N Engl J Med 2008 359 2488 2491 19052131 10.1056/NEJMe0808185

[B30] Page K Morris MD Hahn JA Maher L Prins M Injection Drug Use and Hepatitis C Virus Infection in Young Adult Injectors: Using Evidence to Inform Comprehensive Prevention Clin Infect Dis 2013 57 suppl 2 S32 S38 23884063 10.1093/cid/cit300 PMC3722077

[B31] Treloar C Rance J Backmund M Understanding Barriers to Hepatitis C Virus Care and Stigmatization From a Social Perspective Clin Infect Dis 2013 57 suppl 2 S51 S55 23884066 10.1093/cid/cit263

[B32] De Vos AS Kretzschmar MEE The efficiency of targeted intervention in limiting the spread of HIV and Hepatitis C Virus among injecting drug users J Theor Biol 2013 333 126 134 23733004 10.1016/j.jtbi.2013.05.017

[B33] Des Jarlais DC McKnight C Arasteh K Feelemyer J Perlman DC Hagan H Cooper HLF Transitions from injecting to non-injecting drug use: Potential protection against HCV infection J Subst Abuse Treat 2013 10.1016/j.jsat.2013.09.004 PMC3947032 24161262

[B34] Bruggmann P Litwin AH Models of Care for the Management of Hepatitis C Virus Among People Who Inject Drugs: One Size Does Not Fit All Clin Infect Dis 2013 57 suppl 2 S56 S61 23884067 10.1093/cid/cit271 PMC6279207

[B35] Harris M Rhodes T Hepatitis C treatment access and uptake for people who inject drugs: a review mapping the role of social factors Harm Reduct J 2013 10 7 23651646 10.1186/1477-7517-10-7 PMC3686576

[B36] Toy M Veldhuijzen IK de Man RA Richardus JH Schalm SW Potential impact of long-term nucleoside therapy on the mortality and morbidity of active chronic hepatitis B Hepatol Baltim Md 2009 50 743 751 10.1002/hep.23061 19585616

[B37] Hutton DW Tan D So SK Brandeau ML Cost-effectiveness of screening and vaccinating Asian and Pacific Islander adults for hepatitis B Ann Intern Med 2007 147 460 469 17909207 10.7326/0003-4819-147-7-200710020-00004

[B38] European Centre for Disease Prevention and Control | Hepatitis B and C surveillance report 2006-2011 Stockholm, Sweden http://www.ecdc.europa.eu/en/publications/_layouts/forms/Publication_DispForm.aspx?ID=877&List=4f55ad51%2D4aed%2D4d32%2Db960%2Daf70113dbb90

