# Supplementary material for: Policy responses to viral hepatitis B and C among people who inject drugs in Member States of the WHO European region: a sub-analysis of the WHO 2013 global hepatitis policy survey
Source: BMC Infect Dis. 2014 Sep 19;14(Suppl 6):S15. doi: 10.1186/1471-2334-14-S6-S15 (PMC4178545; doi:10.1186/1471-2334-14-S6-S15)
Supplement: Additional file 1 — Survey instructions and questions selected for this sub-analysis presented with initial survey numbering [file 1471-2334-14-S6-S15-S1.docx]

**Additional file 1:** Survey instructions and questions selected for this sub analysis presented with initial survey numbering

**Responding to Viral Hepatitis: The World Health Organization/World Hepatitis Alliance 2012 Survey of National Governments**

Your cooperation is requested in gathering data that will enable the World Health Organization and the World Hepatitis Alliance to assess the response to viral hepatitis at the global, regional and national levels. This information is vital for measuring progress, identifying gaps and guiding future efforts. Please fully answer all questions, in English. You may provide supporting information in any language. If you do not know the answer to a question, please make every effort to find the information. Please submit the completed survey, along with all supporting information, to [obaran@who.int](mailto:obaran@who.int)with a cc to [hepatitis@cphiv.dk](mailto:hepatitis@cphiv.dk) .

Contact Information

**First name _____________________________________________**

**Last name _____________________________________________**

**Position _______________________________________________**

**Organisation __________________________________________**

**Street address __________________________________________**

**__________________________________________**

**City ___________________________________________________**

**Postal code/zip code _____________________________________**

**Country ________________________________________________**

**Phone number __________________________________________**

**E-mail address ___________________________________________**

**Website _______________________________________________**

**Where appropriate, please provide the names and contact details of colleagues providing supporting information.**

**1. In your country, is there a *written* national strategy or plan that focuses exclusively or primarily on the prevention and control of viral hepatitis?***

**( ) yes ( ) no ( ) do not know**

*** Please e-mail us the relevant supporting information including if you have a strategy or plan for other diseases that includes viral hepatitis.**

**If yes –**

**Is the strategy or plan exclusive for viral hepatitis or does it also address other diseases (e.g. HIV, sexually transmitted infections)?**

1. **( ) exclusive for viral hepatitis**
2. **( ) only for hepatitis B**
3. **( ) only for hepatitis C**
4. **( ) integrated with other diseases**
5. **( ) do not know**

**Please indicate whether the following are components of the strategy or plan:**

| 1. **Raising awareness** | **( ) yes** | **( ) no** | **( ) do not know** |
| --- | --- | --- | --- |
| 1. **Surveillance** | **( ) yes** | **( ) no** | **( ) do not know** |
| 1. **Vaccination** | **( ) yes** | **( ) no** | **( ) do not know** |
| 1. **Prevention in general** | **( ) yes** | **( ) no** | **( ) do not know** |
| 1. **Prevention of transmission of viral hepatitis via injecting drug use** | **( ) yes** | **( ) no** | **( ) do not know** |
| 1. **Health care transmission prevention** | **( ) yes** | **( ) no** | **( ) do not know** |
| 1. **Treatment and care** | **( ) yes** | **( ) no** | **( ) do not know** |
| 1. **Coinfection with HIV** | **( ) yes** | **( ) no** | **( ) do not know** |

**4. Did your government hold events for World Hepatitis Day 2012?***

**( ) yes ( ) no ( ) do not know**

*** Please e-mail us the relevant supporting information.**

**5. Has your government funded any public viral hepatitis awareness campaigns since January 2011, other than World Hepatitis Day?***

**( ) yes ( ) no ( ) do not know**

*** Please e-mail us the relevant supporting information (including the names of the campaigns).**

**If yes, what were the primary topics or messages of the campaigns?**

1. **( ) General information about hepatitis and its transmission**
2. **( ) Vaccination for hepatitis A and hepatitis B**
3. **( ) Importance of knowing one’s hepatitis B and hepatitis C status**
4. **( ) Safe water and good sanitation**
5. **( ) Safer sex practices**
6. **( ) Harm reduction for people who inject drugs**
7. **( ) Safe workplace practices**

**h.( ) Other ­– please specify: a. ________________________________**

**b. ________________________________**

**6. Does your government have a viral hepatitis prevention and control program that includes activities targeting specific populations?**

**( ) yes ( ) no ( ) do not know**

**If yes, please indicate which populations:**

1. **( ) Health workers (including health care waste handlers)**
2. **( ) People who inject drugs**
3. **( ) Migrants**
4. **( ) Prisoners**
5. **( ) The homeless**
6. **( ) People living with HIV**
7. **( ) Low-income populations**
8. **( ) The uninsured**
9. **( ) Indigenous people**
10. **( ) Other ­(please specify): _____________________**

**19. Are viral hepatitis serosurveys conducted regularly?**

**( ) yes ( ) no ( ) do not know**

**If yes –**

- 1. **How often? ____________**
  2. **When was the last one carried out? ____________**
  3. **Please specify the target populations:**
     1. **() Children (please specify age group): ______________________**
     2. **() General population**
     3. **() People who inject drugs**
     4. **() Men who have sex with men**
     5. **() Other groups: _______________________**

**33. Is there a national policy relating to the prevention of viral hepatitis among people who inject drugs?***

**( ) yes ( ) no ( ) do not know**

*** Please e-mail us the relevant supporting information.**

**37. Please answer the following questions about hepatitis B and hepatitis C testing in your country. Please check the appropriate boxes.**

|  | 1. **Hepatitis B** | 1. **Hepatitis C** |
| --- | --- | --- |
| 1. **When testing, do people register by name?** | **() Yes**  **() No** | **() Yes**  **() No** |
| 1. **If people register by name, are their names kept confidential within the system, or is there open access?** | **() Confidential**  **() open access** | **() Confidential**  **() open access** |
| 1. **Is the test free of charge for all individuals?** | **() Yes**  **() No** | **() Yes**  **() No** |
| 1. **Is the test free of charge for members of any specific groups?** | **() Yes (please indicate): ____**  **() No** | **() Yes (please indicate): ____**  **() No** |
| 1. **Is the test compulsory for members of any specific groups?** | **() Yes (please indicate): ____**  **() No** | **() Yes (please indicate): ____**  **() No** |

**39. Is publicly funded treatment available for hepatitis B?**

**( ) yes ( ) no ( ) do not know**

1. **If yes, who is eligible for publicly funded treatment for hepatitis B? Please specify:**

**40. Is publicly funded treatment available for hepatitis C?**

**( ) yes ( ) no ( ) do not know**

1. **If yes, who is eligible for publicly funded treatment for hepatitis C? Please specify:**

**42. Which of the following hepatitis B drugs and hepatitis C drugs are included on the national essential medicines list or are subsidised by the government? (Please check all that apply.)**

1. **Available treatments for hepatitis B:**
2. **( ) Interferon alpha**
3. **( ) Pegylated interferon**
4. **( ) Lamivudine (Epivir-HBV, Zeffix or Heptodin)**
5. **( ) Adefovir dipivoxil (Hepsera)**
6. **( ) Entecavir (Baraclude)**
7. **( ) Telbivudine (Tyzeka, Sebivo)**
8. **( ) Tenofovir (Viread)**
9. **( ) Other: _______________________**
10. **Available treatments for hepatitis C:**
11. **( ) Interferon alpha**
12. **( ) Pegylated interferon**
13. **( ) Ribavirin**
14. **( ) Boceprevir (Victrelis)**
15. **( ) Telaprevir (Incivo, Incivek)**
16. **( ) Other: _______________________**
